# Respiratory-Correlated (RC) vs. Time-Resolved (TR) Four-Dimensional Magnetic Resonance Imaging (4DMRI) for Radiotherapy of Thoracic and Abdominal Cancer

**DOI:** 10.3389/fonc.2019.01024

**Published:** 2019-10-11

**Authors:** Guang Li, Yilin Liu, Xingyu Nie

**Affiliations:** Department of Medical Physics, Memorial Sloan Kettering Cancer Center, New York, NY, United States

**Keywords:** 4DMRI, radiation therapy (radiotherapy), tumor motion assessment, treatment planning and delivery, respiratory motion and motion variation

## Abstract

Recent technological and clinical advancements of both respiratory-correlated (RC) and time-resolved (TR) four-dimensional magnetic resonance imaging (4DMRI) techniques are reviewed in light of tumor/organ motion simulation, monitoring, and assessment in radiotherapy. For radiotherapy of thoracic and abdominal cancer, respiratory-induced tumor motion, and motion variation due to breathing irregularities are the major uncertainties in treatment. RC-4DMRI is developed to assess tumor motion for treatment planning, whereas TR-4DMRI is developed to assess both motion and motion variation for treatment planning, delivery and assessment. RC-4DMRI is reconstructed to provide one-breathing-cycle motion, similar to 4D computed tomography (4DCT), the current clinical standard, but with higher soft-tissue contrast, no ionizing radiation, and less binning artifacts due to the use of an internal respiratory surrogate. Recent studies have shown that its spatial resolution has reached or exceeded that of 4DCT and scanning time becomes clinically acceptable. TR-4DMRI is recently developed with an adequate spatiotemporal resolution to assess tumor motion and motion variations for treatment simulation, delivery and assessment. The super-resolution approach is most promising since it can image any organ/body motion, whereas RC-4D MRI are limited to resolve only respiration-induced motion and some TR-4DMRI approaches may more or less depend on RC-4DMRI. TR-4DMRI provides multi-breath motion data that are useful not only in MR-guided radiotherapy but also for building a patient-specific motion model to guide radiotherapy treatment using an non-MR-equipped linear accelerator. Based on 4DMRI motion data, motion-corrected dynamic contrast imaging and diffusion-weighted imaging have also been reported, aiming to facilitate tumor delineation for more accurate radiotherapy targeting. Both RC- and TR-4DMRI have been evaluated for potential clinical applications, such as delineation of tumor volumes, where sufficiently high spatial resolution and large field-of-view are required. The 4DMRI techniques are promising to play a role in motion assessment in radiotherapy treatment planning, delivery, assessment, and adaptation.

## Introduction

Respiratory motion management is a critical component for radiotherapy of malignant tumors in the thorax and abdomen, including lung, liver, pancreatic, and adrenal cancer. Clinical strategies for motion management include breath-hold, abdominal compression, as well as 4D imaging techniques for targeting internal tumor volume (ITV), respiratory gating, and tumor tracking (1–3). For motion assessment of a mobile tumor, 4D imaging is necessary to provide a patient-specific motion margin for treatment. In image-based treatment planning, respiratory-correlated (RC) four-dimensional computed tomography (4DCT) is the current clinical standard to assess respiratory-induced tumor motion. In image-guided radiotherapy (IGRT) using a conventional medical linear accelerator (Linac), 4D cone-beam CT (4D CBCT) may be applied for patient setup, while periodic MV/kV imaging, intrafractional motion review (IMR), and video-based optical surface imaging may be applied for intrafractional motion monitoring (3). Recently, magnetic resonance imaging (MRI) has been increasingly applied for MR-based planning and MR-guided radiotherapy (MRgRT), and various 4DMRI techniques have been developed, including respiratory-correlated (RC) and time-resolved (TR) 4DMRI.

Similar to 4DCT, RC-4DMRI is reconstructed by binning partial volumetric images or k-space data, such as 2D MR slice images, based on the signal from a respiratory surrogate assuming periodic respiration. However, RC-4DMRI provides higher soft-tissue contrast to visualize gross tumor volume (GTV) and nearby organs at risk (OARs) without ionizing radiation, higher image quality with less binning artifacts by using an internal respiratory surrogate to eliminate the uncertainties from an imperfect external-internal correlation, and higher precision in assessing the primary superior-inferior motion within image slices by imaging in the sagittal or coronal directions. Unlike 4DCT and RC-4DMRI, dynamic or TR-4DMRI does not assume periodic motion because it captures the images of a moving object on the fly. Therefore, TR-4DMRI is ideal to assess respiratory motion, which is often irregular. Furthermore, organ motion can be driven jointly by other involuntary motions, such as cardiac and digestive motions, or voluntary body motion. These motions are either random in nature or having a different rhythm; neither correlates well with respiration. So, the GTV/OAR motion may be complex and non-periodical. Therefore, TR-4DMRI is useful to assess respiratory motion in multi-breathing cycles with irregularities but no binning artifacts, providing higher 4DMRI image quality for GTV/ITV delineation and more realistic GTV/OAR motion for treatment planning and dose delivery assessment.

Historically, dynamic 3D cine MRI was first studied for imaging respiratory motion through direct acquisition (4). This would be the most desirable 4D imaging form to assess organ motion regardless it is regular or irregular, simple or complex, and voluntary or involuntary because it does not assume periodic motion and does not need any respiratory surrogate for reconstruction. However, only low spatial resolution 3D cine was achieved because it was limited by the slow physical MR relaxation and large clinical field of view, even though parallel imaging and view-sharing approximation were applied. Despite the recent development of state-of-the-art MRI techniques (5–7), the basic limit of MR acquisition speed is still present. Facing the fundamental challenge, alternative approaches were developed to circumvent this limitation, including a super-resolution (SR) approach to achieving higher spatiotemporal resolution by combining two sets of MRI image series with complementary strength in either high temporal or high spatial resolution (8, 9). The dynamic 3D cine images acquired with low spatial resolution in free-breathing (FB) of a patient serve as the motion template to map the high spatial resolution from a breath-hold (BH) 3D MRI image of the same patient through deformable image registration (DIR).

In this short review article, we will start with a discussion on RC-4DMRI developments in section Respiratory-Correlated (RC) 4DMRI, with a different emphasis from two recent review articles on the RC-4DMRI (10, 11). The focus of this review will be on different approaches to reconstruct TR-4DMRI, with emphasis on the SR approach and its potentials, and on the discussion and comparison with RC-4DMRI in section Time-Resolved (TR) 4DMRI. The utility of the TR-4DMRI in clinical research and potentials in clinical applications will be discussed in section Clinical Evaluations and Applications of RC- and TR-4DMRI. Finally, we will summarize the recent advancements and provide an outlook for future development in the 4DMRI field.

## Respiratory-Correlated (RC) 4DMRI

Respiratory-correlated 4DMRI consists of several 3D images covering different respiratory states of one breathing cycle of a patient, similar to 4DCT. The similarity and difference among 4DCT, 2D/3D cine MRI, as well as RC- and TR-4DMRI are summarized in Table 1. It is worthwhile to mention that the use of an internal MR navigator as the surrogate reduces binning artifacts because it eliminates the uncertainty from the external-internal correlation if an external surrogate is used (12). In addition, the versatility of MR acquisition and reconstruction allows different ways for sorting the 2D MR slice images: (1) scanning k-space data via Cartesian, Radial, or Spiral acquisition, (2) orienting acquisition in axial, sagittal or coronal directions, (3) binning in image space or k-space, and (4) reconstructing the images prospectively or retrospectively.

**Table 1:** Comparison of RC-4DMRI, 2D/3D cine MR, TR-4DMRI with 4DCT and 2D kV/MV.

**Category**	**Sub-category**	**4DCT**	**RC-4DMRI**	**2DkV/MV**	**2D cine**	**3D cine**	**TR-4DMRI**
Acquisition	Scanning	Projection	2D slice	Projection	2D slice	3D volume	3D
	Moving Couch	Yes	No	No	No	No	No
	Ionizing Radiation	Yes	No	Yes	No	No	No
	Preferred Scan Directions^∧^	Axi	Axi/Sag/Cor	Obl/Sag/Cor	Obl/Sag/Cor	Sag/Cor	Sag/Cor
	3D Recon^#^	FBP	iFFT	NA	NA	iFFT	iFFT
	4D Recon^#^	Binning	Binning	NA	NA	NA	SR
Contrast	Lung^&^	High	High (T2)	Low	Mid	Mid	High (T2)
	Abdomen^&^	Low	High	Low	High	High	High
Motion	Respiratory Surrogate	External	Internal (/External)	NA	NA	NA	NA
	Cyclical Motion Assumption	Yes	Yes	No	No	No	No
	Binning Artifacts^$^	High	Low	No	No	No	No
	Multi-breathing Cycles	No	No	Yes	Yes	Yes	Yes

∧ *Preferred scan directions include Axial (Axi), Sagittal (Sag), Coronal (Cor), and oblique (Obl). The coronal scan is often used in 3D cine due to shorter anterior-posterior separation of the human body*.

# *Reconstruction methods using the filtered back project (FBP), inverse fast Fourier transform (iFFT), and super-resolution (SR) methods*.

& *T1 or balanced steady-state free-precession (bSSFP) MR contrasts are used for real-time scan*.

$ *High binning artifacts for irregular breathers in 4DCT. Low binning artifacts in RC-4DMRI when using an internal navigator with the Cartesian acquisition and No binning artifacts when using self-navigator in the Golden-angle radial acquisition*.

In a prospective approach, the acquisition intends to fill the table with the row of respiratory state and the column of image slice. When all the table elements are acquired, the acquisition and reconstruction of RC-4DMRI are completed. Hu et al. developed a prospective T2-weighted 4D-MRI method with a respiratory amplitude-based triggering system to gate 2D MRI image acquisition (13). Either an internal or external surrogate can be applied, but the former produced superior image quality than the latter (12). In a retrospective approach, similar to 4DCT scan, more scans than a cycle may be acquired to reconstruct complete 3D images in all respiratory states. Cai et al. proposed a T2/T1-weighted 4DMRI retrospective phase sorting method that used body area extracting from images as the respiratory surrogate (14). Van de Lindt et al. reported a self-sorting coronal 4DMRI technique for MR-Linac (15).

The reconstruction of RC-4DMRI in k-space can also be categorized as either prospective or retrospective. Akçakaya et al. developed a 4DMRI k-space respiratory gating method using an internal navigator as the surrogate to prospectively gate the acquisition of the central k-space data (16). Liu et al. developed a strategy that retrospectively reorders k-space data of MR images based on respiratory trajectories, allowing for finer segmentation of data in the time domain (17).

Recently, efforts have been devoted to making RC-4DMRI scan more efficient with higher image resolution and quality. Feng et al. developed a golden-angle radial acquisition technique with compressed sensing (CS) using local self-navigator(s) to resolve both respiratory and cardiac motions in RC-4DMRI (or 5D MRI) (18–20). Because of using radial acquisition, which is insensitive to motions, the RC-4DMRI images are essentially free from binning artifacts. Golden-angle acquisition and multi-navigators facilitate the CS scheme of reconstruction to resolve both respiratory and cardiac motions. Similarly, Wang et al. reported a spatiotemporal k-space scan and sorting technique to enhance RC-4DMRI image quality (21). Han et al. reported a rotating Cartesian k-space (ROCK) 4DMRI method that provides a 50 × 40 × 30 cm^3^ field of view, 1.2 × 1.2 × 1.6 mm^3^ voxel size, 8 respiratory states, and 5 min scan time (22, 23). This method was equivalent to spiral acquisition and was validated with a 4D phantom and tested in volunteers and patients, producing 1.0 ± 0.5 mm error at the diaphragm in comparison with 2D cine image. Mickevicius and Paulson compared 4 different 4DMRI image reconstruction algorithms and found that the two with CS outperformed the conventional techniques (24). A similar conclusion was reported by Weiss et al. comparing conventional and CS-based liver scans using navigator-gated 4DMRI acquisition with a 1.2 × 1.2 × 3.0 mm^3^ resolution (25). Van Reeth et al. studied the proof of concept of an SR approach to achieve isotropic image resolution by combining anisotropic scans (26). Freedman et al. also developed an SR approach to gain T2w RC-4DMRI with an isotropic resolution (1.0 × 1.0 × 1.0 mm^3^) by combining 2 acquisitions in axial and sagittal scans at 1.5 × 1.5 × 5.0 mm^3^ resolution (27).

So far, the image resolution is approaching or exceeding that of 4DCT, while the binning artifacts have been substantially reduced or virtually eliminated and scanning time has become clinically acceptable. With the advantages of soft-tissue contrast enhancement and elimination of ionizing radiation, RC-4DMRI technique has been increasingly tested in the clinic and have the potential to play a role in clinical applications (10, 11, 28, 29). However, RC-4DMRI shares the same limitation of one breathing cycle as 4DCT, while using a snapshot of patient respiration as the average motion has been questioned for its validity and reliability (30–32).

## Time-Resolved (TR) 4DMRI

**Figure 1:**
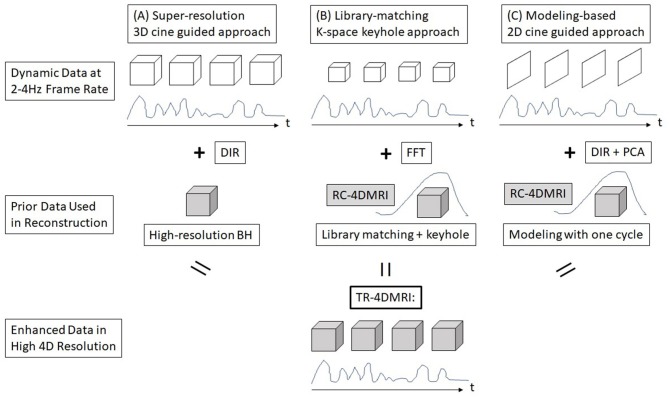
A schematic graph of three reconstruction methods for time-resolved (TR) 4DMRI. **(A)** Super-resolution (SR) approach, **(B)** dynamic keyhole approach, and **(C)** motion modeling approach.

Breathing irregularity has been recognized as a major clinical issue in radiotherapy because it causes a tumor to move differently from the motion assessment achieved at treatment simulation, so that the treatment delivery may not follow the treatment plan. Clinically, it has been observed that substantial breathing irregularities may occur, leading to a possible large variation in tumor motion (30–32). In addition, patient breathing irregularities during motion simulation may affect RC-4DMRI image quality, which further affects tumor delineation, although it has been improved using internal navigator as the respiratory surrogate (12). On the contrary, TR-4DMRI provides multi-breath motion assessment and may immune from breathing irregularities if it is based on SR reconstruction. The major advantages of TR-4DMRI are summarized in Table 1. As direct acquisition of 3D cine suffers from low spatial resolution, three alternative methods have been developed to reconstruct TR-4DMRI with a clinically-adequate spatiotemporal resolution. Taking the advantage of 4D patient anatomy redundancy, incomplete but near real-time scans are sufficient to recover the missing information from *a priori* knowledge of the patient for TR-4DMRI reconstruction, as illustrated in Figure 1. It should be emphasized that only the SR approach (Figure 1A) is independent of RC-4DMRI without assuming motion periodicity, while the other two approaches depend on RC-4DMRI in various degrees for library building (Figure 1B) or motion modeling (Figure 1C).

### Super-Resolution (SR) 3D-Cine-Guided TR-4DMRI Reconstruction

Super-resolution is a concept that has been proven effective to enhance the resolution of an imaging modality beyond its physical limitation (33). It achieves this objective by combining two image sets with complementary resolution strength using an independent method so that both strengths will appear in the final synthesized image. The SR concept was applied to overcome the physical limitation of dynamic 3D cine MRI (8).

Li et al. reported an SR approach to reconstruct TR-4DMRI by combining two sets of MRI images with high temporal resolution (3D cine at 2 Hz and 5 × 5 × 5 mm^3^) in free-breathing (FB) and high spatial resolution in breath-hold (BH, at 2 × 2 × 2 mm^3^) through DIR (8), as shown in Figure 1A. Therefore, the resulting TR-4DMRI image will have a high spatiotemporal resolution (2 Hz and 2 × 2 × 2 mm^3^). In this approach, the dynamic FB 3D cine serves as targeting templates for DIR to map the high-resolution tissue texture from BH to FB images. Because the FB image records the actual organ motion without any restriction on the motion type, so this approach can image both regular or irregular organ motions, including breathing irregularities in multiple respiratory cycles. This method has been improved with enhanced deformation range for respiratory motion (9). Furthermore, the MR contrasts can be extended beyond T1w, including T2w TR-4DMRI (34). Therefore, the SR-based TR-4DMRI technique is promising to provide the accurate history and statistics of actual GTV/OAR motions during treatment simulation and/or treatment delivery.

### Library-Matching Dynamic Keyhole TR-4DMRI Reconstruction

The dynamic keyhole method is derived from the conventional keyhole approach, a view-sharing technique, which divides the k-space into the central (low frequency) and peripheral (high frequency) regions, where only the central data need to be newly acquired and updated while the peripheral data can be acquired separately and shared. The dynamic keyhole method requires anatomical matching between the central and peripheral k-space data so that the aliasing artifacts caused by anatomy mismatch can be minimized (35, 36).

Lee et al. reported a dynamic keyhole method by image matching at the moving diaphragm in a pre-acquired high-resolution RC-4DMRI library (37, 38). A 1D keyhole was applied to combine the central and peripheral k-space data to produce a dynamic TR-4DMRI image set. Liu et al. studied the dynamic volumetric keyhole method as a k-space SR approach for accelerated TR-4DMRI reconstruction without DIR (39). The RC-4DMRI image was applied to create the motion library with high-resolution, however the library can also be created by the TR-4DMRI images. Therefore, limited dependency of this approach to the RC-4DMRI exists. The major advantage of this approach is the fast reconstruction comparing with DIR-based reconstruction.

Comparing with CS, the keyhole method is inferior in both image quality and acceleration (36). Using statistical power in resolving sparse, incoherent signals is superior to mechanically-separated high- and low-frequency regions in the k-space. Yip et al. incorporated a dynamic view-sharing technique into the CS framework and developed a sliding-window prior-data-assisted CS (SW-PDACS) technique to track lung tumor motion (40, 41). In this approach, the k-space was divided into 3 regions, central, middle and peripheral regions for random sampling covering different parts of the k-space, allowing partial k-space update continuously. Therefore, dynamic cine can be acquired with reduced sampling and reconstructed by view sharing with acceptable image quality.

### Model-Based 2D-Cine-Guided TR-4DMRI Reconstruction

A third method to reconstruct TR-4DMRI image is based on a motion model that is built on the RC-4DMRI and dynamic 2D cine to guide the model to deform to provide 4D volumetric images. Therefore, this method is fully dependent on RC-4DMRI. Dynamic 2D cine has been utilized for motion assessment and MR-guided radiotherapy on an MR-Linac (MRL) system (42, 43). The frame rate of 4 Hz is available in current commercial systems, although recent studies have shown that the temporal resolution can be further improved (6, 44).

Although 2D cine images are insufficient for volumetric motion assessment of GTV and OAR for treatment planning or treatment delivery, the missing volumetric data can be obtained from prior 4DMRI scans of the same patients. Harris et al. reported a method to retrieve the volumetric information from RC-4DMRI by building a patient-specific respiratory motion model, which can be deformed using the dynamic 2D cine as the guidance (45). This method requires RC-4DMRI, DIR, and principal component analysis (PCA) to build the motion model, similar to a method that was developed in 4DCT (46). Stemkens et al. reported the same technique of TR-4DMRI but focusing on abdominal tumor (47) and 4DMRI can be also used for dynamic contrast-enhanced (DCE) imaging for better tumor delineation (48).

Other 2D cine-guided reconstruction methods of TR-4DMRI were also reported under certain clinical conditions and assumptions. Paganelli et al. acquired interleaved orthogonal 2D cine images, deformed them to an RC-4DMRI library, and extrapolated the 2D displacement vector fields (DVFs) for 3D image reconstruction (49). When deformation is small, 2D cine may be close to the same phase as a 3D image within volumetric RC-4DMRI (50). Park et al. used local rigid registration in a small region of interest that contains the tumor to retrieve the tumor motion to study internal-external motion correlation (51). The method served the purpose of extracting the tumor motion, but it may inherit a higher degree of uncertainty for full-image reconstruction, especially for motion that does not correlate well with respiration.

## Clinical Evaluations and Applications of RC- and TR-4DMRI

High soft-tissue contrast in RC-4DMRI facilitates tumor/organ delineation and registration for treatment planning. Zhang et al. reported organ segmentation based on T2w RC-4DMRI (28), including the heart, lungs, liver, and stomach in 10 volunteers and evaluated manual and DIR-propagated organ segmentation using STAPLE algorithm. A 95% confident-level ground truth was created to quantify the quality of individual contour with specificity, sensitivity, and Jaccard index. The DIR-propagated contours were found as good as human contours owing to the high soft-tissue contrasts in T2w 4DMRI. Zhang et al. also reported lung tumor delineation in 10 patients from 6 radiation oncologists and compared the results with those from 4DCT and T1w breath-hold images (29). All images were acquired on the simulation day within 2 h. It was found that T2w RC-4DMRI produced a similar GTV to 4DCT but with a much smaller variation among physicians, while T1w MRI based GTV is about 25% smaller. In addition, the tumor motion variation can be quite large, leading to a very different ITV. Gao et al. studied an accelerated 4DMRI for treatment planning (44). An abdominal tumor and two kidneys were delineated and compared between two acceleration imaging techniques with quantification of positioning, volume difference, Dice similarity index and mean distance to the agreement. Liu et al. explored the 4D diffusion-weighted imaging (DWI) MRI imaging for tumor delineation (52). A 4D digital phantom and volunteers were tested for the feasibility. The clinical workflow for RC-4DMRI has been investigated for MR-only treatment planning since only one-cycle motion is provided, like 4DCT (53), by converting MR voxel intensity to tissue electronic density for dose calculation and generating digitally-reconstructed radiography (DRR) with visualized fiducials for patient setup (43).

Assessment of radiotherapy treatment has also been performed using a TR-4DMRI technique since it records exactly what happens during the beam-on time in MRL treatment. Thomas et al. reported the patient intra- and inter-fractional motion variations using dynamic 2D cine during 3-5 fraction SBRT treatments (30). Mostafaei et al. studied that the localization of the gallbladder is affected by both respiratory and peristaltic motions (32). The 2D cine images can be converted to volumetric TR-4DMRI using the DIR-PCA approach (Figure 1C) for retrospective treatment evaluation. Kontaxis et al. reported a strategy to perform online intrafraction replanning for free-breathing stereotactic body radiation therapy using MRL (54). The dosimetry consequence of motion variation from treatment planning viewpoint was studied using RC- and TR-4DMRI, in comparison with the ITV method based on 4DCT (55). Substantial dosimetry variations in ITV-based planning were found when the tumor motion range varied by 5 mm, and this is worthwhile for further evaluations.

In scanning proton therapy, motion interplay effect was found substantial (56) and respiratory-gated proton therapy may be a viable solution (57). Dolde et al. applied five repeated RC-4DMRI to simulate and evaluate motion variation and dose delivery in proton therapy (57). It was also found that the residual error of 2-3 mm in DIR has a large impact on dose assessment owing to the high dose conformality with a sharp dose falloff outside of the target for most proton plans (58).

For patient motion simulation, Stam et al. reported using 2D cine MRI to characterize kidney motions in FB (59). Park et al. reported that using an external surrogate to predict an internal tumor motion may suffer from its insensitivity to internal motion variation and a phase mismatch (51). Wilms et al. reported using multivariate regression approaches for diffeomorphic estimation of internal tumor motion based on surrogates (60). Milewski et al. reported a large phase shift between the external and internal motion based on the internal navigator echo (1D cine) and bellows data during FB 4DMRI acquisition and successfully enhanced the external-internal motion correlation by correcting the phase shift (61). Interestingly, the phase shift was found to be relatively stable over 7–13 min despite breathing irregularities. These studies could lead to the development of a robust patient-specific motion model for respiratory gating in the clinic.

TR-4DMRI provides actual patient breathing motion images over multiple breathing cycles, and therefore serves as an imaging tool for real-time motion monitoring in MRL and provides motion data for building a patient-specific breathing motion model for tumor motion prediction in non-MRL systems. Clinically, the scanning time of TR-4DMRI is determined by how much patient motion statistics is needed. This technique suggests that dynamic 3D/2D cine can be converted to volumetric TR-4DMRI with adequate spatiotemporal resolution for more clinical evaluations, including GTV and OAR motion and motion variations for radiotherapy treatment planning and delivery. An MR simulator or an MRL provides simulation or treatment motion images, which are useful for treatment planning, assessment of treatment delivery, and building a patient-specific multi-breath motion model.

The technical development and clinical application of RC- and TR-4DMRI are currently at their infancy and require further explorations to fully realize their potentials in radiotherapy with new clinical workflows. Like any other techniques, the RC- and TR-4DMRI have their own limitations, which may lead to future research for improvements. For RC-4DMRI, the major limitations are a single breathing cycle, long acquisition time, and minor MR image distortion, while image resolution has been substantially improved to reach or exceed that of 4DCT. For TR-4DMRI, the major limitations include spatial image uncertainty and image reconstruction time owing to the DIR-based method, while the k-space or GPU-based reconstruction approaches may provide viable solutions. In clinical applications, physician training on target delineation based on 4DMRI images is essential to fully realize the value of the new techniques, because most radiation oncologists are trained for CT-based target delineation. Currently, dedicated MR simulators are only available in a few academic institutions but not in community clinics/hospitals, while MRL machines are even fewer in the world. Regardless of the dimension and form of MRI images, radiotherapy applications require reliable conversion from MR voxel intensity to tissue electron density for accurate radiation dose computation, especially for MR-only treatment planning. Despite these technical and financial limitations, RC- and TR-4DMRI are promising to play a role in radiotherapy because the unparallel ability to differentiate tumorous tissue from its surrounding normal tissues and to image patient continuously without motion periodicity assumption.

## Summary and Future Perspective

Both RC- and TR-4DMRI have been developed in recent years and continued to be further studied in the future, especially for high spatial-resolution RC-4DMRI with reduced binning artifacts and high spatiotemporal-resolution TR-4DMRI with improved reconstruction. These 4DMRI techniques have been and will be evaluated under clinical conditions for applications, including tumor/organ delineation and motion assessment for treatment planning and treatment dose evaluation. These allow online or offline adaptive planning and treatment, using 4DMRI-based assessment of the delivered dose to a mobile tumor and surrounding OARs. High soft-tissue contrast and high spatial resolution of RC-4DMRI are useful to improve clinical target delineation, especially in the central thoracic and abdominal regions where 4DCT suffers from the inability to distinguish a tumor from surrounding healthy tissues. Because of the one-cycle motion image of RC-4DMRI similar to 4DCT and the fact of 5 years in advance of TR-4DMRI development, RC-4DMRI is more likely to be first applied to the clinic in the near future.

The main advantage of TR-4DMRI over RC-4DMRI is that it provides multi-breath motion images during the patient simulation and/or treatment. As common breathing irregularities may cause large uncertainties in radiotherapy, the TR-4DMRI technique can serve as a tool to assess and monitor motion irregularities in MRL, aiming to improve treatment accuracy. In light of MRL development, there is a strong clinical need to further develop TR-4DMRI and to make it clinically available to prospectively guide and retrospectively assess radiation therapy treatment. Based on the multi-breath motion data from the simulation, a patient-specific motion model can be built that would be useful to provide intrafractional tumor motion guidance for non-MR Linac systems. With more representative motion assessment from TR-4DMRI, respiratory gating or tumor tracking could reliably be applied so that the motion margin can be reduced, resulting in more OAR sparing and thus allowing more potent dose prescription to treat a mobile tumor.

## Author Contributions

GL wrote the final version of the article. YL and XN participated drafting the initial version.

### Conflict of Interest

The MSKCC has a master research agreement (MRA) with Philips Healthcare. The authors declare that the research was conducted in the absence of any commercial or financial relationships that could be construed as a potential conflict of interest. The handling editor declared a past supervisory role with one of the authors YL.
